# Long-term outcome prediction using IMPACT and APACHE II in patients with traumatic brain injury treated in the ICU

**DOI:** 10.1186/cc13664

**Published:** 2014-03-17

**Authors:** R Raj, R Kivisaari, J Siironen, M SkrifVars

**Affiliations:** 1 Helsinki University Central Hospital, Helsinki, Finland

## Introduction

The International Mission for Prognosis and Analysis of Clinical Trials (IMPACT) is currently the most robust prognostic model in patients with traumatic brain injury (TBI). No studies have compared this TBI-specific prediction model with general ICU models, such as the Acute Physiology and Chronic Health Evaluation II (APACHE II) [1,2]. This study investigates the performance and the correlation of the IMPACT and APACHE II models for long-term outcome prediction in patients with TBI.

## Methods

The study population consisted of TBI patients admitted to a designated ICU in southern Finland during a 4-year period (2009 to 2012). The IMPACT and APACHE II performances were assessed by calculating discrimination (by area under the curve), calibration (by GiViTI calibration test and Hosmer-Lemeshow *C *statistic) and precision (by Brier score). Correlation between the IMPACT and APACHE II was tested by Spearman's rho. Primary outcome was 6-month mortality.

## Results

**Figure 1:**
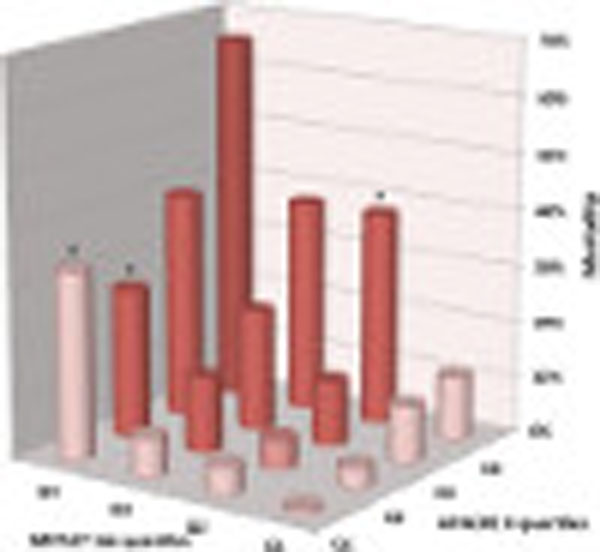
Area under the curve for the new models.

Of the total 897 included patients, overall 6-month mortality was 22%. The IMPACT and APACHE II showed similar AUCs (0.81, 95% CI = 0.77 to 0.84; 0.80, 95% = CI 0.76 to 0.83) and Brier scores (0.134, 0.135). Calibration tests revealed significant (*P *< 0.05) deviations between observed and predicted risk for both models. A moderately strong, positive correlation between the IMPACT and the APACHE II was noted (Spearman's rho = 0.566, *P *< 0.001). The IMPACT and the APACHE II were found to identify slightly different groups of patients that eventually do not survive (Figure 1).

## Conclusion

The IMPACT and the APACHE II models showed equal performance for 6-month mortality prediction. A moderately strong, positive correlation, with some major discrepancies between the models, was found. Thus, features of both the IMPACT and APACHE II models are valuable for optimal outcome prediction in patients with TBI treated in the ICU.
